# Migration of endothelial cells into photo-responsive hydrogels with tunable modulus under the presence of pro-inflammatory macrophages

**DOI:** 10.1093/rb/rbz025

**Published:** 2019-07-30

**Authors:** Wangbei Cao, Xuguang Li, Xingang Zuo, Changyou Gao

**Affiliations:** MOE Key Laboratory of Macromolecular Synthesis and Functionalization, Department of Polymer Science and Engineering, Zhejiang University, Hangzhou, China

**Keywords:** 3D cell migration, immune-response, hydrogel, photo-responsive, hyaluronic acid

## Abstract

Cell migration in three-dimensional environment is extremely important for tissue regeneration and other biological processes. In this work, a model system was developed to study how endothelial cells (ECs) migrate into photo-responsive hydrogels under the presence of pro-inflammatory macrophages. The hydrogel was synthesized from hyaluronic acid grafted with coumarin and methacrylate moieties by both carbon–carbon covalent linking and coumarin dimerization under UV irradiation at 365 nm. The structure of the hydrogel was conveniently modulated by UV irradiation at 254 nm to decompose the coumarin dimers, leading to the significant decrease of modulus and increase of swelling ratio and mesh size. Under the presence of M1 macrophages, ECs were induced to migrate into the hydrogels with a different degree. A significant larger net displacement of ECs was found in the softer hydrogel obtained by irradiation with UV at 254 nm than in the stiffer original one at day 7.

## Introduction

Cell migration is referred to the movement of cells in response to specific external signals including biochemical factors and changes in extracellular matrix (ECM) [1]. As one of the fundamental cellular functions, cell migration in three-dimensional (3D) environment plays an important role in biology processes such as angiogenesis [2], tissue regeneration [3] and inflammation [4]. Therefore, control over the cell migration is of both significance in fundamental studies and design of biomaterials with better performance for tissue regeneration. One of the creative ways to achieve this goal is to regulate the inflammatory response after biomaterials are implanted by applying different phenotypes of macrophages [5–7]. Traditionally macrophages are only considered as a kind of pro-inflammatory and destructive cells [8]. Currently, macrophages of different phenotypes have been recognized, including the pro-inflammatory phenotype (M1, the ‘classic phenotype’), and the anti-inflammatory/regulatory phenotypes (M2) [9]. The M1 phenotype induced by interferon-γ (IFN-γ) and lipopolysaccharide (LPS) can secrete cytokines such as interleukin-1β (IL-1β), tumor necrosis factor-α (TNF-α) and vascular endothelial growth factor (VEGF) [10]. The M2 phenotype induced by IL-4, IL-10 and IL-3 can secrete cytokines such as matrix metalloprotease-9 (MMP-9), transforming growth factor-β (TGF-β), and platelet-derived growth factor-BB (PDGF-BB) to accelerate the process of tissue regeneration. Both types of macrophages can induce cell migration in a different way [11].

In most cases *in vivo*, cells are located in a 3D and dynamic environment, implying that cells would interact three-dimensionally with their matrix which is changing over time [12]. In this regard, the stimuli-responsive materials with tunable properties, which are also called ‘intelligent materials’, are ideal model systems for investigating cell migration in order to simulate the dynamic behaviors *in vivo*. The structures and properties of stimuli-responsive materials can be changed by various environmental stimuli [13] such as pH [14], light [15], redox [16], temperature [17] and enzymes [18]. In particular, the light-responsive materials have been widely studied in biomedical applications including anti-bacteria [19] and drug delivery [20] because of the versatilities in light wavelength, intensity, location and time [21]. Among the intelligent materials, intelligent hydrogels are more promising since their mechanical properties, microstructures and high water content are similar with human tissues [22–24], and are definitely interacted with cells in a 3D manner. Moreover, hydrogels made of nature-originated polymers such as alginate, chitosan, collagen, hyaluronic acid (HA) and gelatin have been widely used as biomaterials due to their biomimetic structures and high bioactivity [25].

HA is a kind of glycosaminoglycan whose chains are composed of glucuronic acid and *N*-acetyl-glucosamine. It exists ubiquitously in all kinds of mammals in a salt form as hyaluronate in the synovial fluid around joints, cartilage and tissues of eyes as vitreous body and skin [26]. Due to its extremely good biocompatibility and great potential for chemical modification [27], HA has been widely used in many biomedical fields.

**Scheme 1:**
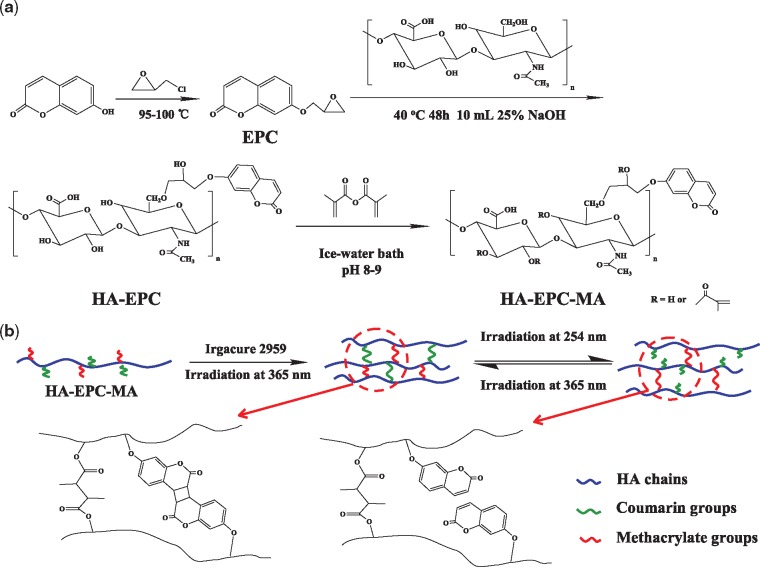
(A) schematic illustration to show the synthetic procedures of the photo-responsive HA-EPC-MA molecule. (B) The crosslinking of HA-EPC-MA by both covalent bonds and reversible coumarin dimerization, and thereby the modulation of crosslinking density by UV irradiation at 254 and 365 nm. In this process, the integrity of the hydrogel can be maintained

In this work, the cell migration in photo-responsive hydrogels with different stiffness is studied for the first time under the presence of pro-inflammatory cells (macrophage M1), which exist and interact ubiquitously for any implanted biomaterials. The light-responsive hydrogel is adopted as the matrix because its structures and properties could be conveniently modulated by light *in situ* without adding any other reagent. The hydrogel precursor is synthesized by modifying HA with methacrylate and coumarin moieties (HA-EPC-MA) (Scheme 1). The crosslinking formed by the methacrylate moieties is insensitive to environmental stimuli and thus can maintain the integrity of hydrogel, whereas the dimerized coumarin moieties formed at 365 nm can be easily decomposed by UV irradiation at 254 nm [28], leading to the modulation of the crosslinking density. Endothelial cells (ECs) are cultured atop the hydrogels either irradiated by UV at 254 nm or not, which are placed into the upper Boyden chamber, while the pro-inflammatory macrophages are seeded on the well of a culture plate. The pro-inflammatory macrophages can secrete cytokines such as TNF-α, inducing the migration of ECs into the hydrogels. The migration distance and net displacement are quantified and compared in the HA hydrogels of different crosslinking density.

## Materials and methods

### Materials

7-Hydroxy-coumarin (Umbelliferone, Co., Ltd., USA) was purchased from Sigma-Aldrich Co., Ltd. Epichlorohydrin (AR), potassium hydroxide (AR), sodium hydroxide (AR), chloroform (AR), anhydrous sodium sulfate (AR) and *N*,*N*-dimethylformamide (DMF, AR) were purchased from Sinopharm Chemical Reagent Co., Ltd (China). HA (Mn = 100 kDa) was purchased from Zhenjiang Dongyuan Biotech Co., Ltd. (China). Methacrylic anhydride (MA) was purchased from Aladdin Chemical Co., Ltd. LPS (from *Escherichia coli* O111: B4) was purchased from Sigma-Aldrich Co., Ltd. IFN-γ was purchased from PROSPEC Co., Ltd. Sprague–Dawley rats (120 g) were bought from Zhejiang Academy of Medical Science. Recombinant human macrophage colony stimulating factor (MCSF) was purchased from Peprotech PeproTech, Inc. The transwell molds and 24-well culture plates were bought from Corning Co., Ltd., USA. Fetal bovine serum (FBS) was purchased from Sijiqing Inc., China.

### Preparation of photo-responsive hydrogels

#### Synthesis of epoxypropoxy coumarin

7-Hydroxy-coumarin (3.24 g) was dissolved in 100 ml ethanol, into which 5 ml 25% (w/v) KOH solution was added. After the mixture was stirred for 30 min at room temperature, 20 ml epichlorohydrin was added. The solution was refluxed under agitation at 95°C for 2.5 h. Then the solvents were removed by rotary evaporation at 55°C. The residue was dissolved in a mixture of MilliQ water (80 ml) and chloroform (100 ml), which was transferred into a 250 ml separating funnel. The organic phase was collected. After the extraction process was repeated twice, the organic phase was dehydrated by anhydrous sodium sulfate overnight. The raw product was obtained after filtration and rotary evaporation, which was purified by recrystallization in 100 ml ethanol to yield the white solid epoxypropoxy coumarin (EPC).

#### Modification of HA-EPC

About 1 g HA (Mn = 100 kDa) was dissolved in 90 ml MilliQ water, into which 10 ml 25% (w/v) NaOH solution was added. After being stirred for 30 min at room temperature, 20 ml 12.5% (w/v) EPC/DMF solution was added dropwise slowly with continuous agitation. The reaction was maintained for 48 h at 40°C. The mixture was then transferred into a 250 ml separating funnel containing 80 ml chloroform to allow sufficient mixing. After 2 h, the inorganic phase was extracted, and the process was repeated twice. The inorganic solution was dialyzed against deionized water at room temperature for 7 days (MWCO = 3500 g/mol), and the HA-EPC was obtained via vacuum lyophilization.

#### Synthesis of photo-responsive hydrogel precursor (HA-EPC-MA)

About 0.5 g HA-EPC was dissolved in a mixed solvent of DMF (15 ml) and water (30 ml), into which 4 ml MA was added dropwise. The pH of the mixture was adjusted at 8–9 by adding 5 M NaOH solution. After the reaction was maintained in an ice-water bath for 24 h, the mixture was precipitated in 800 ml cold ethanol to obtain the raw product. The raw product was dissolved in MilliQ water and dialyzed against deionized water at room temperature for 7 days (MWCO = 3500 g/mol). The final HA-EPC-MA was obtained via vacuum lyophilization.

#### Preparation of the photo-responsive hydrogel

About 75 mg HA-EPC-MA was dissolved in 1 ml 0.1% (w/v) Irgacure 2959 photoinitiator solution to obtain 7.5% (w/v) HA-EPC-MA solution. About 150 μl of the solution was injected into a mold with an inner diameter of 7.2 mm. The hydrogel was prepared by exposing the mold to UV irradiation (INTELLI-RAY 400, Uvitron, 100% of density or 400 W/cm^2^) for 30 min to crosslink the C=C and coumarin structures.

To study the change of structures and properties of the hydrogels, some of the hydrogels were exposed to UV light (*λ* = 254 nm, 6 W, Shanghai Jiapeng Technology Co., Ltd.) for 10 min on both top and bottom sides. The hydrogels treated by 254 nm UV light were compared with those untreated as described below.

### Characterizations

The ^1^H NMR spectra were recorded on Burker DMX-500 using tetramethylsilane as the internal standard to verify the grafting degree of EPC and MA on HA. EPC was dissolved in CDCl_3_, and HA-EPC and HA-EPC-MA were dissolved in D_2_O at a concentration of 10 mg/ml.

FTIR spectra were used to distinguish 7-hydroxy-coumarin and EPC, which were mixed with KBr separately to prepare samples. The spectra were recorded by using a Vector 22 spectrophotometer (Bruker optics, Switzerland).

To characterize whether the hydrogel could be formed by the dimerization of coumarin, 0.15 g HA-EPC was dissolved in 2 ml MilliQ water, and 150 μl of this solution was injected to 48-well plate. Then the solution was exposed to UV light (*λ* = 365 nm) for 30 min. The hydrogel was then exposed to UV light (*λ* = 254 nm) for 20 min to decompose the coumarin dimers. Digital images were taken to show the sol–gel transition.

The morphology, swelling ratio and mechanical properties of the HA-EPC-MA hydrogels before and after exposed to UV light (*λ* = 254 nm) were measured to show the changes of structure and properties.

The morphology of hydrogels was examined by using a scanning electron microscope (SEM, Hitachi S-4800). The hydrogels were washed with MilliQ water three times and then lyophilized under –20°C, which were then cut to show their inner structures, and coated with a thin layer of gold before characterization. The diameter of the pores was analysed by the ImageJ software. At least 100 pores were randomly chosen and measured.

The weights of the lyophilized hydrogels before (*W*_d_) and after (*W*_s_) incubation in MilliQ water for 24 h were measured, which were used to calculate the swelling ratio by (*W*_s_ – *W*_d_)/*W*_d_. Each value was averaged from three parallel samples.

Compressive modulus was measured by a mechanical tester (Instron 5543A) at a rate of 0.2 mm min^−1^. In the compressive curve, the *Y* coordinate is related to the value of compressive stress while the *X* coordinate is related to the strain. The compressive modulus was calculated by the slope of the compressive curve when the strain was below 5%. Three parallel samples were measured to calculate the average values.

### 3D migration of ECs into photo-responsive

The Sprague–Dawley rats were used to extract macrophages (M_0_) with an enterocoelia injection method according to a previous protocol [11]. 3 × 10^5^ extracted cells were cultured per well when 24-well culture plate was used. Each well contained 1 ml DMEM, in which the final concentrations of 2-mercaptoethanol, streptomycin, penicillin and FBS were 0.05 mM, 100 µg/ml, 100 U/ml and 10%, respectively. The culture conditions for these cells were controlled at 37°C and 5% CO_2_. Then, a fresh medium with 300 ng/ml LPS, 50 ng/ml IFN-γ and 20 ng/ml MCSF was added to induce the polarization of the macrophages. The macrophages were cultured in the same fresh medium for the following experiments after they were polarized for 48 h.

Human vein ECs were obtained from the Cell Bank of Typical Culture Collection of Chinese Academy of Sciences (Shanghai, China). The ECs were cultured in high-glucose DMEM, in which streptomycin, penicillin and FBS were added, with the same final concentrations and conditions mentioned above. After about 3 days, the cells were detached and subcultured.

To monitor the migration of ECs into the photo-responsive hydrogels, the hydrogels were divided into two groups based on whether being exposed to UV light (*λ* = 254 nm) or not. Before cell seeding and insertion into the Boyden chamber, they were incubated in PBS conditioned with complete DMEM for 2 h. 3 × 10^5^ pro-inflammatory macrophages (M1) were seeded per well of a 24-well culture plate with 500 μl medium. Then the Boyden chamber with a hydrogel was inserted into the well with macrophages. 200 μl medium including 3 × 10^5^ ECs were then planted atop each hydrogel to observe the migration behaviors. In a total period of 7 days, fresh medium was renewed twice with the constant stimulators for the macrophages. The whole system was maintained at 5% CO_2_ and 37°C for 1 or 7 days.

The migration depth of ECs was measured by confocal laser scanning microscopy (CLSM, ZEISS LSM780, Germany). The cells-seeded scaffolds were washed three times with PBS at pre-determined time intervals. Then the ECs were fixed for 1 h with 3.9% paraformaldehyde/PBS solution, following with washes for three times. After being permeabilized for 5 min at 4°C by 0.1% Triton-X100, the samples were washed for three times again, and then maintained for 1 h with 1% BSA/PBS. Finally, DAPI (dilution 1:100) and rhodamine–phalloidin (dilution 1:400) were used to stain the ECs in the scaffolds for 1 h at 37°C. By using CLSM with a 20× objective lens, a series of images in *Z*-axis with a 5-μm step were taken under the excitation wavelengths of 405 and 561 nm. Then the *Z*-axis series images were reconstructed to the whole images by using the ZEN software. The depth of ECs and migration pattern could be measured and recorded by the two different channels (blue for nuclei and red for cytoskeleton). Three parallel samples were measured to obtain mean values of migration depth.

### Statistical analysis

The experimental data are expressed as mean ± standard deviation with three parallel samples, and were analysed by one-way analysis of variance for statistical analysis. The statistical significance was set as *P* ≤ 0.05.

## Results and discussion

### Synthesis and characterization of HA-derivatives

**Figure 1:**
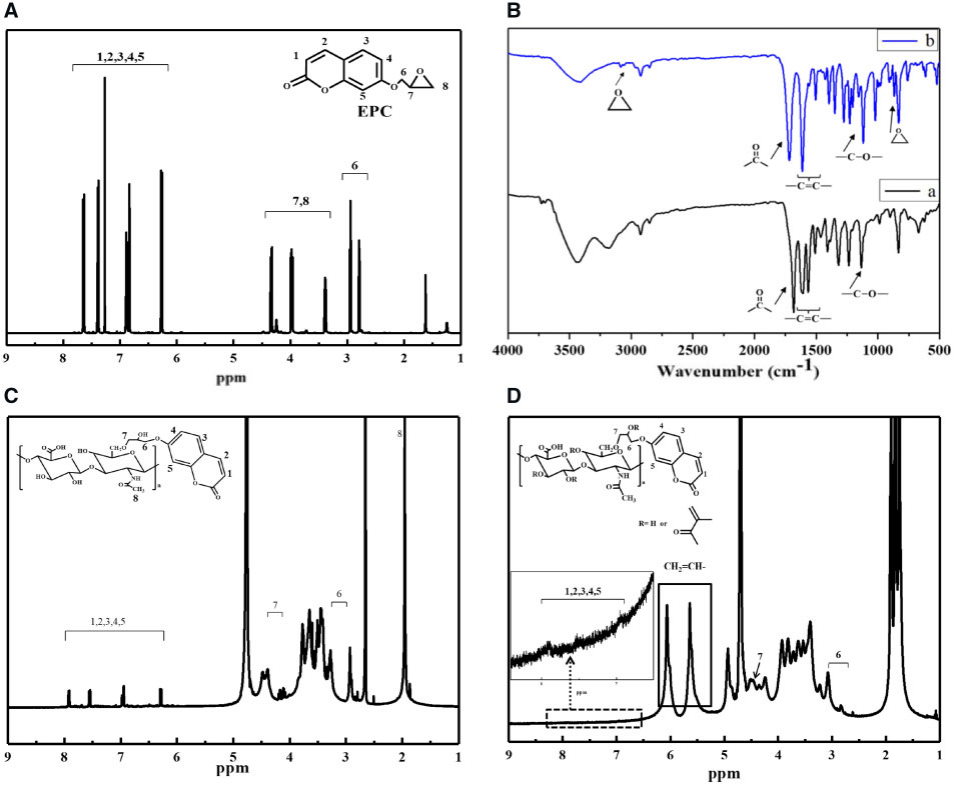
(A) ^1^H NMR spectrum of EPC. (B) FTIR spectra of (a) 7-hydroxycoumarin and (b) EPC. (C) ^1^H NMR spectrum of HA-EPC. (D) ^1^H NMR spectrum of HA-EPC-MA. Molecular structures or characteristic groups are shown inside the figures

7-Hydroxy-coumarin was reacted with epichlorohydrin to synthesize EPC via the substitution reaction (Scheme 1A), whose structure was confirmed by ^1^H NMR spectroscopy (Fig. 1A) (500 MHz, CDCl_3_) [29]. The peaks at 6.25, 6.8, 6.9, 7.4 and 7.65 ppm were referred to the protons of coumarin structure, and the peaks of protons in the epoxy structure appeared at 3.4, 4.0 and 4.35 ppm. The –CH_2_O– groups between the coumarin and epoxy structure appeared at 2.8 and 2.95 ppm. Integral of the peak intensity found that the aromatic ring of coumarin structure and epoxy structure was about 1:1, proving that the –OH group of 7-hydroxy-coumarin was completely substituted by the epoxy structure. The molecular structures of 7-hydroxy-coumarin and EPC were further characterized by FTIR spectroscopy (Fig. 1B). The major absorption peaks of 7-hydroxy-coumarin are assigned as follow: –OH stretching, 3174.5 cm^−1^; C=O group, 1694.5 cm^−1^; C=C, 1506.0–1612.4 cm^−1^; C–O, 1133.9 cm^−1^. After epoxy substitution, the absorption of –OH stretching disappeared, and the absorptions assigned to epoxy group appeared at 868.3 and 3090 cm^−1^. The yield of EPC was 63.1%.

The EPC-modified HA was synthesized by the ring-opening addition reaction between epoxy group of EPC and –OH groups of HA in alkaline environment (Scheme 1A). The molecular structure of EPC-HA was confirmed by ^1^H NMR spectroscopy (Fig. 1C). The peak at about 2.0 ppm was referred to the CH_3_CO– groups in the HA backbone. The peaks at 6.28, 6.94, 6.98, 7.55 and 7.91 ppm demonstrated the presence of aromatic ring in the coumarin structure. The peaks of –CH_2_O near coumarin structure appeared at 2.79 and 2.92 ppm, and the peaks of –CH_2_O near HA chains appeared at 4.10 and 4.16 ppm. If the peak intensity of the CH_3_CO– groups in HA backbone was normalized as 1, the peak intensity of hydrogens in the aromatic ring of coumarin structure was calculated as 0.2 by the Mestnova Software, revealing that the molar ratio of EPC groups and acetyl groups in EPC-HA was 0.12, i.e. the grafting degree was 12%.

**Figure 2:**
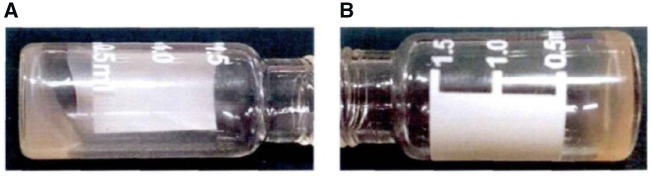
Titled vials containing HA-EPC (A) before and (B) after irradiation at 365 nm

The EPC-HA could be readily crosslinked to form a hydrogel as shown in Fig. 2. After exposed to UV irradiation (*λ* = 365 nm) for 30 min, the 7.5% (w/v) HA-EPC solution (Fig. 2A) was transformed into a hydrogel (Fig. 2B), demonstrating the occurrence of dimerization of the grafted coumarin groups. The formed hydrogel could be decomposed after irradiated by UV (*λ* = 254 nm) for 20 min to form the EPC-HA solution, demonstrating the applicability of reverse change of coumarin monomer and dimer.

The photo-responsive hydrogel precursor HA-EPC-MA was synthesized by the condensation reaction of hydroxyl groups of HA-EPC and MA (Scheme 1A). Its structure was confirmed by ^1^H NMR spectroscopy too (Fig. 1D). The peaks at 5.64 and 6.06 ppm were referred to the two asymmetric hydrogen atoms on the carbon–carbon double bonds, confirming the successful synthesis of HA-EPC-MA. The peak at 1.95 ppm is assigned to the methyl group of HA backbone, and the peaks at 1.75 and 1.84 ppm are assigned to MA. The resonance peaks of aromatic ring of coumarin structure appeared at 6.94, 7.52 and 7.91 ppm (theoretically, there was a peak at 6.28 ppm, but was overlapped with the peaks of the asymmetric hydrogen atoms). The –CH_2_O groups near coumarin structure appeared at 2.76 and 2.93 ppm, and the peak of –CH_2_O groups near HA appeared at 4.34 ppm. By calculating the area ratio of the peaks above, the grafting degree of C=C was calculated as 48% (based on each repeating unit of HA). The grafting degree of EPC was not changed.

### Characterization of HA-based photo-responsive hydrogels

**Figure 3:**
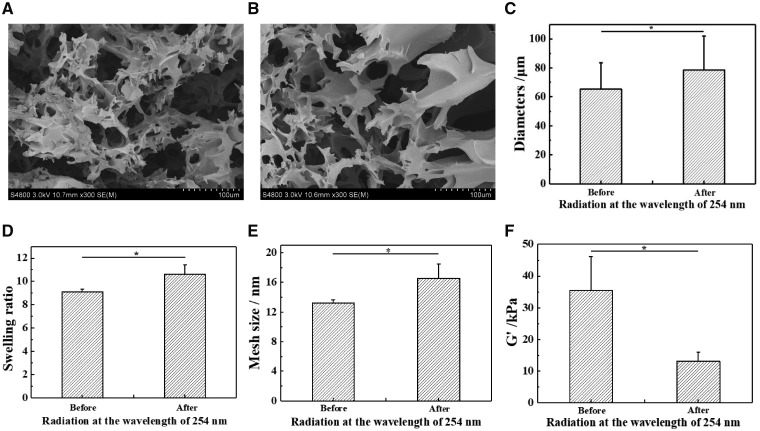
SEM images of photo-responsive hydrogels (A) before and (B) after irradiation at the wavelength of 254 nm. (C) Pore size of dry hydrogels before and after irradiation at *λ* = 254 nm. (D) Swelling ratio, (E) mesh size and (F) elastic modulus of photo-responsive hydrogels before and after irradiation at *λ* = 254 nm

The HA-EPC-MA molecules are easily to form hydrogels via covalent linking between the carbon–carbon double bonds and dimerization of coumarin (Scheme 1B). This dimerization degree of coumarin could be adjusted by UV irradiation time at *λ* = 365 nm [30–32]. In this study a sufficient long irradiation (30 min) was applied to enable the largest degree of crosslinking of carbon–carbon double bonds and coumarin dimerization. Then the coumarin dimers were decomposed by UV irradiation at 254 nm to adjust distinctly the mechanical modulus of the resulted hydrogels (Scheme 1B). In this way, the overall crosslinking degree of the hydrogels could be adjusted conveniently, while the integrate structure could still be kept due to the irreversible crosslinking of carbon–carbon double bonds. Figure 3A and B show the microstructure of freeze-dried hydrogels before and after being irradiated by UV light (*λ* = 254 nm). They both had a porous structure regardless of the UV irradiation, which is typical for the lyophilized hydrogels. However, quantitative analysis by the Origin software reveals that the average diameter of the pores was enlarged significantly from 65.2 ± 18.3 to 78.6 ± 23.3 μm after UV irradiation (Fig. 3C). It has to mention that the apparent pore size measured in a dry state was 3 magnitude larger than the mesh size of hydrogels measured in a wet state (Fig. 3E), and was influenced by many processing parameters. Nevertheless, the decomposition of coumarin dimers leads to a smaller crosslinking density of the hydrogels, and thereby the weaker strength and poorer ability to restrict the growth of ice crystals at low temperature, forming larger ice particles which correspond to the pores after lyophilization.

Moreover, the swelling ratio of the hydrogels was significantly increased too (Fig. 3D), demonstrating the decrease of crosslinking density. According to Flory-Huggins theory, the mesh size (*M_c_*) can be calculated:

$$\frac{M_{c}\left(\frac{1}{2}-\chi_{1}\right)}{\rho_{2}V_{1}}=Q^{5/3}$$

where *ρ*_2_ is the density (g/cm^3^) of the polymers, *χ*_1_ is the Huggins parameter which reflects the change of interaction between polymers and solvent, *V*_1_ is the molar volume of water (18 ml/mol) and *Q* is the volume swelling ratio of polymers in equilibrium (*Q* is positively related to the weight swelling ratio in this experiment). In this experiment, water was used as a good solvent for dissolving HA, so the *χ*_1_ is <1/2. Therefore, the mesh size in the polymer networks increased after decomposition of the coumarin dimers (Fig. 3E).

The elastic modulus of the hydrogels was measured by a compression test at room temperature. Corresponding to the decrease of crosslinking density, the modulus decreased significantly from 35.4 ± 10.7 to 13.1 ± 2.9 kPa after UV irradiation at 254 nm (Fig. 3F).

All these results prove that the HA-EPC-MA hydrogels could maintain their macroscopic structures after UV irradiation at 254 nm, but their crosslinking density and elastic modulus were weakened to some extend due to the decomposition of the coumarin dimers.

### Migration of ECs into the HA-EPC-MA hydrogels

**Figure 4:**
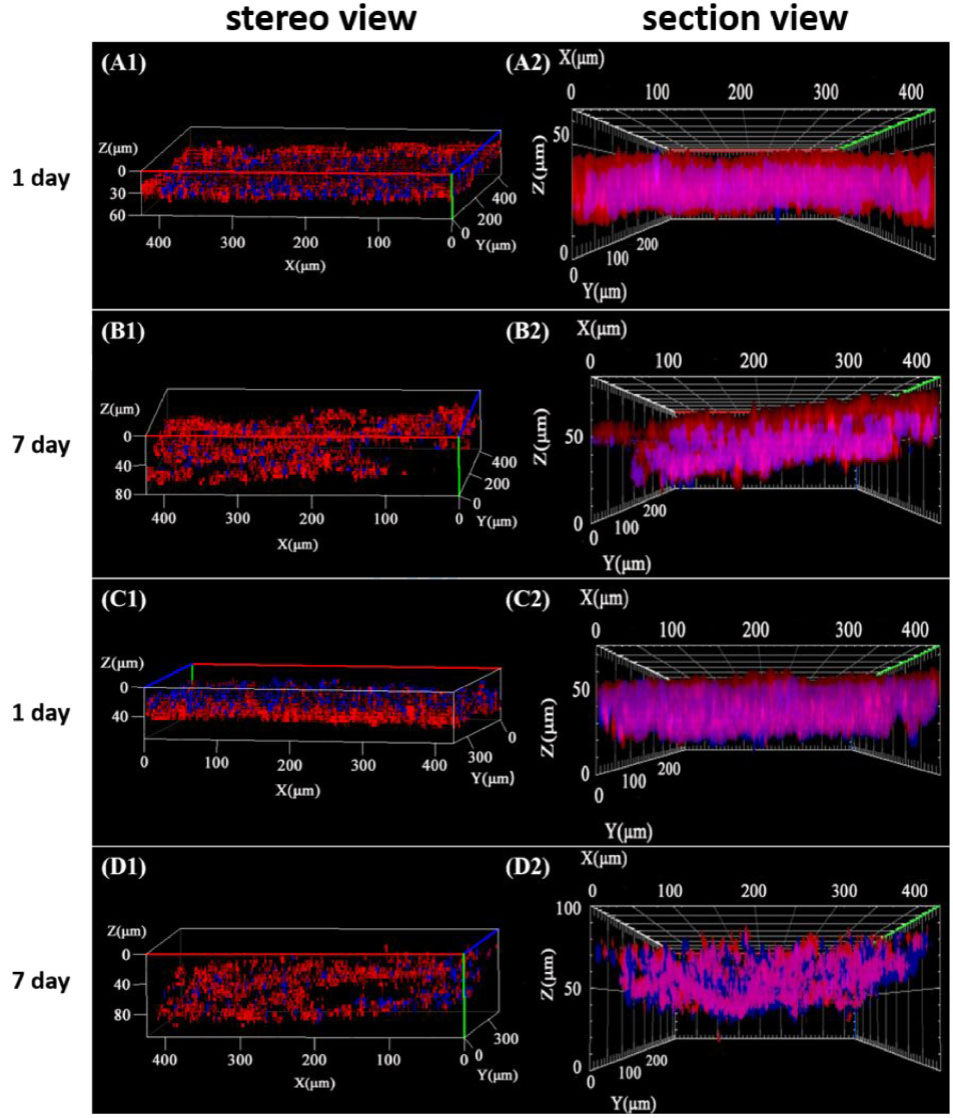
(A1)–(D1) Stereo view and (A2)–(D2) section view of 3D-reconstructured confocal images of ECs after being cultured for (A, C) 1 day and (B, D) 7 days atop photo-responsive hydrogels (A, B) without and (C, D) with irradiation at *λ* = 254 nm, respectively. Under the lower well of Boyden chamber, macrophages polarized to M1 phenotype were seeded

**Figure 5:**
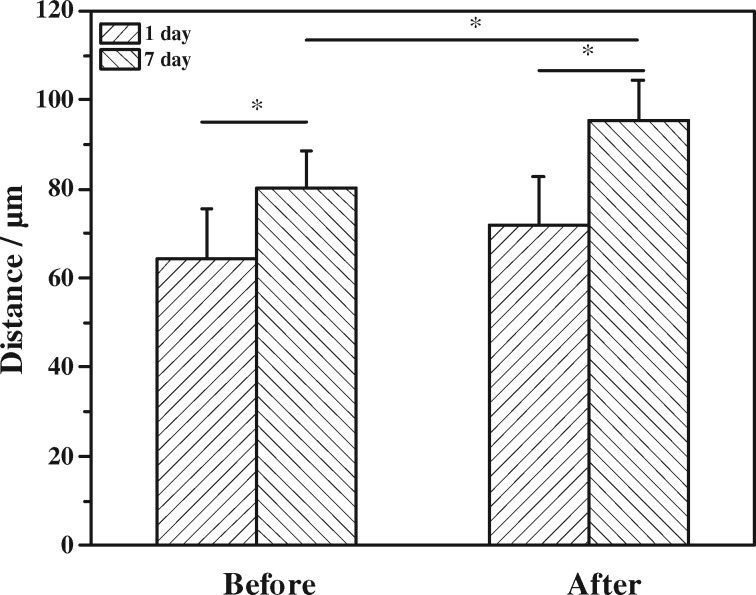
Migration depth of ECs into the hydrogels before and after irradiation at *λ* = 254 nm after being cultured atop the hydrogels for 1 and 7 days

It is known that the migration of cells can be induced by chemical signals such as TNF-α and VEGF secreted by the M1 phenotype macrophages [11]. The macrophages used in this study were polarized into M1 phenotype by 50 ng/ml IFN-γ and 300 ng/ml LPS as reported previously [11]. At this condition, the positive ratio of M1 was about 30%. Our previous study confirmed that the migration depth of cells without macrophages or other types of immune cells would be significantly slower [11, 18]. They were then seeded on the culture well to induce the migration of ECs into the photo-responsive hydrogels which were placed onto the Boyden chamber. The cytoskeletons and nuclei of ECs in the hydrogels were stained and observed by CLSM [18]. The cytoskeletons and cell nuclei were observed as red and blue, respectively (Fig. 4), showing that the ECs were closely packed in the hydrogel matrix. The section views reveal obviously the cell distribution in *Z* direction of the hydrogels. In the original hydrogel without UV irradiation (*λ* = 254 nm), the migration distance reached to 80.4 ± 8.0 μm at day 7, implying the net displacement was about 16 μm compared with the initial value at 1 day (Fig. 5). In the UV (254 nm)-irradiated hydrogel, the initial cell invitation value at 1 day was similar to that in the hydrogel without UV treatment. After culture for 7 days, however, the depth of migration reached to 95.4 ± 9.1 μm with a net displacement of 24 μm which was significantly larger than that in the original hydrogel (Fig. 5). Therefore, the ECs migrated deeper into the UV-irradiated hydrogels, whose crosslinking degree and modulus were decreased due to the decomposition of coumarin dimers. That the ECs migrated more effectively into the materials with a relatively smaller modulus is consistent with previous observation when cells are cultured on substrates with different modulus, including PDMS and hydrogels materials [33–35]. The faster migration of ECs is beneficial for the angiogenesis during the process of tissue regeneration [36–38] due to the easier transport of proangiogenic factors, which in turn promote the migration of ECs [39].

In this study, we set up a new model system *in vitro* to study how ECs migrate in photo-responsive hydrogels under a chemokine gradient formed *in situ* by pro-inflammatory macrophages. We chose coumarin groups as the photo-responsive structure in the hydrogels so that the crosslinking degree of the hydrogels could be adjusted reversibly, which could mimic the dynamic changes of ECM. The solution of HA-EPC-MA molecules was exposed to UV irradiation of 365 nm for 30 min with 0.1% Irgacure 2959 (photoinitiators). Although the time for irradiation was slightly longer and the concentration of photoinitiators was higher than most other studies [40–43], the resulted hydrogels could well maintain their biological activities [44–46]. At such conditions, the carbon–carbon double bonds and the coumarin molecules on the HA chains could achieve the largest reaction extent, and thus the physical properties could be changed as large as possible upon further irradiation by UV light at 254 nm.

It is known that the hydrogels prepared with different degrees of substitution (DS) of MA and coumarin would have different physical properties [32, 44]. The DS of methacrylate and coumarin groups within the HA polymer backbone were fixed as 48% and 12% in this study, respectively. It was not the purpose to adjust the modulus of hydrogels with different values because this is not new and the interactions of cells with hydrogels are rather complicated and thus difficult to evaluate broadly. The structures and properties of hydrogels used in this type of studies can be optimized in the future. Moreover, a 2D tube formation assay in hydrogels may provide more insightful information in terms of angiogenesis [47, 48]. Nevertheless, the use of stimuli-responsive hydrogels moves forward a new step, and the current study provides a useful model and experimental evidence for the design and optimization of responsive biomaterials with better performance in regenerative medicine.

## Conclusions

Cell migration in inflammatory environment is of great significance to mimic the implantation of biomaterials *in vivo*, providing using information on the induced migration of cells to wounds or biomaterials. A new model was developed to study ECs migration in photo-responsive hydrogels under the presence of pro-inflammatory macrophages. The photo-responsive HA hydrogel was prepared by covalent crosslinking of carbon–carbon double bonds and dimerization of coumarin moieties under the UV irradiation at 365 nm. Its crosslinking degree and compressive modulus were decreased by UV light at 254 nm while its integrity was unaffected. The ECs were induced to migrate into the hydrogels under the presence of M1 macrophages. The net displacement of ECs was significantly larger in the hydrogel pre-irradiated by UV at 254 nm compared in the original one, which possessed a larger degree of crosslinking and thereby modulus. Therefore, the crosslinking degree and network structure of the hydrogels are of great importance in regulating the 3D migration of ECs. With the optimization of the compositions and structures of hydrogels, it is possible to modulate the hydrogel properties by light *in situ* and thereby the cell migration behaviors and tissue regeneration process under a more controllable way.

## Funding

Natural Science Foundation of China (51873188, 21434006).

*Conflict of interest statement*. None declared.
